# TQ inhibits hepatocellular carcinoma growth *in vitro* and *in vivo* via repression of Notch signaling

**DOI:** 10.18632/oncotarget.5362

**Published:** 2015-09-21

**Authors:** Xiquan Ke, Yan Zhao, Xinlan Lu, Zhe Wang, Yuanyuan Liu, Mudan Ren, Guifang Lu, Dan Zhang, Zhenguo Sun, Zhipeng Xu, Jee Hoon Song, Yulan Cheng, Stephen J. Meltzer, Shuixiang He

**Affiliations:** ^1^ Department of Gastroenterology, the First Affiliated Hospital, School of Medicine, Xi'an Jiaotong University, Xi'an, Shaanxi 710061, P.R. China; ^2^ Department of Gastroenterology, Tongji Hospital, Tongji University School of Medicine, Shanghai 200065, P.R. China; ^3^ Department of Gastroenterology, Xi'an Central Hospital, Xi'an, Shaanxi 710000, P.R. China; ^4^ Department of Thoracic Surgery, Provincial Hospital Affiliated to Shandong University, Jinan, Shandong 250021, P.R. China; ^5^ Department of Gastroenterology, the Second Affiliated Hospital, Zhejiang University School of Medicine, Hangzhou, Zhejiang 310009, P.R. China; ^6^ Division of Gastroenterology, Departments of Medicine and Oncology and Sidney Kimmel Comprehensive Cancer Center, The Johns Hopkins University School of Medicine, Baltimore, MD 21287, USA

**Keywords:** thymoquinone, hepatocellular carcinoma, Notch, cell cycle, apoptosis

## Abstract

Thymoquinone (TQ) has been reported to possess anti-tumor activity in various types of cancer. However, its effects and molecular mechanism of action in hepatocellular carcinoma (HCC) are still not completely understood. We observed that TQ inhibited tumor cell growth *in vitro*, where treatment with TQ arrested the cell cycle in G1 by upregulating p21 and downregulating cyclinD1 and CDK2 expression; moreover, TQ induced apoptosis by decreasing expression of Bcl-2 and increasing expression of Bax. Simultaneously, TQ demonstrated a suppressive impact on the Notch pathway, where overexpression of NICD1 reversed the inhibitory effect of TQ on cell proliferation, thereby attenuating the repressive effects of TQ on the Notch pathway, cyclinD1, CDK2 and Bcl-2, and also diminishing upregulation of p21 and Bax. In a xenograft model, TQ inhibited HCC growth in nude mice; this inhibitory effect *in vivo*, as well as of HCC cell growth *in vitro*, was associated with a discernible decline in NICD1 and Bcl-2 levels and a dramatic rise in p21 expression. In conclusion, TQ inhibits HCC cell growth by inducing cell cycle arrest and apoptosis, achieving these effects by repression of the Notch signaling pathway, suggesting that TQ represents a potential preventive or therapeutic agent in HCC patients.

## INTRODUCTION

Hepatocellular carcinoma (HCC) is the sixth most frequently diagnosed cancer and the third most common cause of cancer death worldwide. An estimated 748,300 new cases and 695,900 cancer deaths occurred from HCC in 2008. Half of these cases and deaths were estimated to occur in China [1]. HCC incidence rates are increasing rapidly in many parts of the world, including the United States and Europe [2–3]. Potentially curative treatments for early-stage HCC include surgical resection, liver transplantation, and ablation. However, high rates of tumor recurrence after resection or ablation constitute a major challenge, with recurrence rates as high as 70% at 5 years [4]. Palliative therapy for intermediate-advanced HCC includes transarterial chemoembolization (TACE) and sorafenib [5–7], but curative effect is difficult to achieve. Therefore, effective treatment agents are urgently needed in this disease.

**Figure 1:**
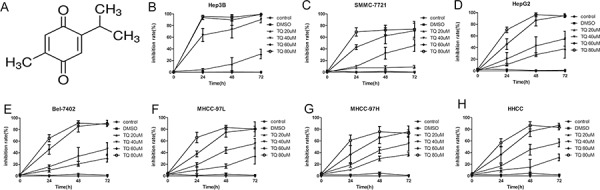
TQ inhibits proliferation HCC cell lines **A.** Chemical structure of TQ. **B-H.** Cell inhibition rate after TQ treatment relative to the controls. HCC cells were cultured in 96-well plates with ascending doses of TQ (20–80 μM) for 24, 48 and 72 h, cell viability was evaluated by MTT assay, the inhibition rate was calculated by formula: (1-treated group/control group) × 100%.

*Nigella sativa* is a widely distributed plant in Mediterranean countries, Pakistan and India, whose seed (black cumin) has been commonly used as a spice, as well as in preservatives and medications, over the past several thousand years; this seed has biological activities including anti-inflammatory, anti-tumor, antioxidant, anti-platelet, anti-atherosclerotic, and anti-diabetic effects [8]. *Nigella* has demonstrated clinical therapeutic activity in many diseases, including bronchial asthma, diarrhea, headache, gastrointestinal illnesses, eczema, hypertension, and obesity [9]. Moreover, there are no obvious adverse effects of this substance. Thymoquinone (TQ), or 2-isopropyl-5-methyl-1, 4-benzoquinone, is the most active monomer isolated from black cumin, with a defined chemical structure (Fig. 1A). Many studies demonstrate that TQ exhibits a broad antitumor activity spectrum [10–13].

There are four transmembrane Notch proteins (Notch 1–4) in mammals [14] and five ligands: Jagged (Jagged 1, 2) and Delta-like (DLL-1, 3, 4), belonging to two protein families [15]. Activation of Notch signaling requires linking of the Notch receptor to its respective ligand in a strictly controlled fashion. The Notch intracellular domain (NICD) is released after Notch receptors undergo a series of proteolytic cleavages [16–17], and NICD then translocates into the nucleus to modulate the expression of Hes1 [18], Bcl-2 [19], and other target genes, which mediate cell proliferation, differentiation and apoptosis, processes that are key to the development and progression of cancer [20–24].

TQ has anti-neoplastic effects on a variety of human cancer cells [10–13]. Recent preliminary reports showed that TQ inhibits HCC growth *in vitro* [25], but potential molecular mechanisms involved in this antitumor effect, in particular its impact on the Notch pathway, along with antitumor effects *in vivo*, have not been studied. Therefore, the present study was conducted to explore the effects and mechanisms of action of TQ against HCC growth *in vitro* and *in vivo*.

## RESULTS

### TQ inhibits the growth of human HCC cells *in vitro*

We examined the effect of TQ on HCC cell proliferation in several HCC-derived cell lines (Hep3B, SMMC7721, HepG2, Bel7402, MHCC97-L, MHCC97-H and HHCC) by MTT assay. Cells with different degrees of differentiation were treated with 20, 40, 60 and 80 μM TQ. TQ significantly inhibited the proliferation of these tumor cell lines in a dose- and time-dependent manner. This anti-proliferative impact was observed within a 24-hour period, and it continued to increase over the next 72 hours (Fig. 1B-1H).

### Notch1 expression in human HCC cell lines and normal liver cells

**Figure 2:**
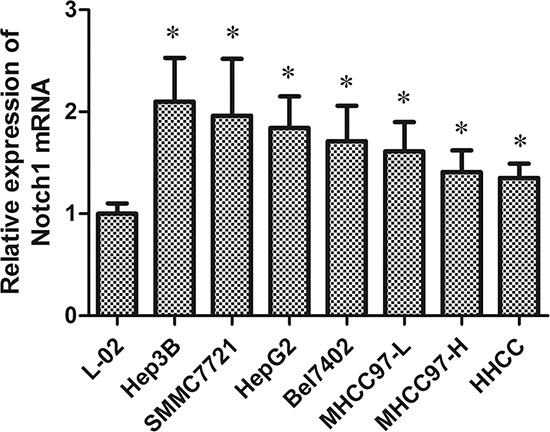
Notch1 mRNA activity in seven HCC cell lines and a normal liver cell for qRT-PCR *p < 0.05.

To explore whether Notch1 is expressed in HCC cells, qRT-PCR analysis was performed in seven HCC cell lines and in primary normal liver epithelial cells (L-02). All seven HCC cell lines expressed higher levels of Notch1 mRNA than did L-02 cells (*P* < 0.05). Hep3B and SMMC7721 expressed the highest levels of Notch1 mRNA among the HCC cell lines tested (Fig. 2). We therefore chose Hep3B and SMMC7721 for further research.

### TQ induces cell cycle arrest by upregulating p21 and downregulating cyclinD1 and CDK2 expression

**Figure 3:**
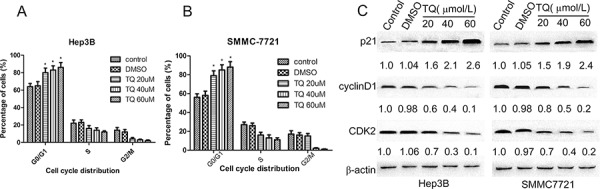
TQ induces G1 phase cell cycle arrest; p21, cylinD1 and CDK2 protein expression in Hep3B and SMMC7721 cells Cells were incubated with various concentrations of TQ for 48 h. Cell cycle distribution was analyzed by flow cytometry. **A.** Proportion of cell population in Hep3B; **B.** cell distribution in SMMC7721. Results are represented as the means ± SE (*n* = 3), *p < 0.05 vs control and DMSO group. **C.** Expression of cell cycle-related protein (above-mentioned) and β-actin proteins was determined by western blot analysis.

To investigate whether inhibition of cell proliferation was associated with cell cycle arrest, we performed flow cytometry to analyze cell cycle distribution after treatment with TQ for 48 h. As shown in Fig. 3A-3B, Hep3B cells treated with TQ (20, 40, and 60 μM) demonstrated an increase in the proportion of cells in G0/G1 phase (79 ± 5.1, 85 ± 5.5, 88 ± 5.9) *vs*. control (0 μM) or DMSO-treated cells (56 ± 3.8 or 58 ± 4.6, respectively), as well as a decrease in the proportion of cells in S phase (16 ± 3, 13 ± 3.2, 11 ± 2.1) *vs*. control or DMSO-treated groups (27 ± 2.9 or 26 ± 2.8, respectively). In SMMC7721 cells, TQ also induced G1-phase arrest in a concentration-dependent manner. To clarify the mechanism of G1 accumulation induced by TQ, we measured expression levels of the cell cycle-related proteins p21, cyclinD1, and CDK2 using Western blot analysis. Results showed that p21 was significantly upregulated, while cyclinD1 and CDK2 were significantly downregulated in a concentration-dependent manner following 48 h of TQ treatment (Fig. 3C).

### TQ induces cell apoptosis and regulates the expression of Bcl-2 and Bax

**Figure 4:**
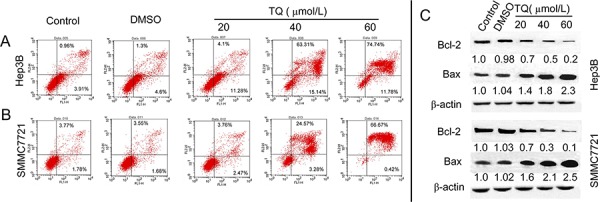
TQ induced cell apoptosis and effect on apoptosis-related factors Cells were stained with AnnexinV and PI, following the quantitative analysis of apoptosis in **A.** Hep3B cells and **B.** SMMC7721 cells by flow cytometry. **C.** TQ decreases expression of Bcl-2 and increases Bax level, with Bcl-2 and Bax measured using western blot analysis.

In addition to cell cycle arrest, we assumed that inhibition of cell growth induced by TQ treatment could also have been caused by programmed cell death. Therefore, we investigated whether TQ induces apoptosis. We measured the number of apoptotic cells after treatment with TQ for 48 h and observed that apoptotic cells were dramatically increased in TQ-treated (20, 40, and 60 μM) Hep3B cells (15.38 ± 3.78, 76.45 ± 7.4, 86.52 ± 8.1) vs. control and DMSO-treated cells (4.87 ± 1.32, 5.9 ± 2.1), while apoptotic cells numbered (6.23 ± 1.07, 27.85 ± 5.67, 67.09 ± 6.97) in TQ-treated (20, 40, and 60 μM) SMMC7721 cells and (5.55 ± 1.39, 5.23 ± 2.04) in control and DMSO-treated SMMC7721 cells, respectively (Fig. 4A-4B). To further investigate the molecular mechanisms underlying these pro-apoptotic effects, we measured expression of the apoptosis-related proteins Bcl-2 and Bax using Western blotting. TQ decreased the expression of Bcl-2 and increased the expression of Bax proteins (Fig. 4C).

### The Notch pathway is involved in the anti-cancer effect of TQ on human HCC cells

**Figure 5:**
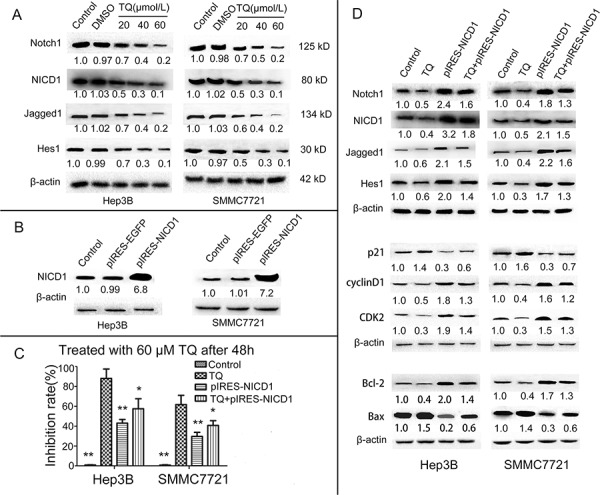
TQ inhibits HCC cells growth by inducing cell cycle arrest and cell apoptosis via the inactivation of Notch pathway genes **A.** Effect of TQ on the Notch pathway, protein levels of Notch1 and NICD1 and Jagged1 as well as Hes1 were determined by western blot. **B.** Verification of Overexpression NICD1 vector, expression of NICD1 in Hep3B and SMMC7721 after transfection with the vector for 48 h was examined using western blot. **C.** Impact of NICD1 overexpression NICD1 on TQ-induced inhibition of the growth of HCC cells. MTT assays were carried out to assess the anti-proliferative effect of TQ on 4 groups of cells; ‘pIRES-NICD1’ indicates the group transfected with a pIRES-NICD1-EGFP vector, *p < 0.05 and **p < 0.01 *vs.* TQ-treated group. **D.** Effect of NICD1 cDNA transfection with or without combination TQ treatment on activity of Notch and its downstream target gene; expression of Notch pathway(Notch1, NICD1, Jagged1, Hes1) and cell cycle-related (p21, cyclinD1 and CDK2) as well as cell apoptosis-related (Bcl-2 and Bax) proteins was measured by western blot analysis.

We hypothesized that TQ-induced growth suppression of HCC cells could be related to the Notch signaling pathway. To confirm this possibility, we determined the effects of TQ on Notch signaling in Hep3B and SMMC7721 cells. Western blotting demonstrated that TQ inhibited the expression of Notch1, NICD1, Jagged1 and Hes1 in a concentration-dependent manner (Fig. 5A). To further elucidate the role of Notch1 in the TQ response, we transfected Hep3B and SMMC7721 cells with pIRES-NICD1-EGFP vector, expressing human NICD1. Initial testing verified that transfection of these cells with this vector successfully and significantly promoted overexpression of NICD1 (Fig. 5B). We then explored whether the inhibitory effect of TQ on cell growth and expression of cell cycle-related and apoptosis-related proteins was related to suppression of Notch signaling. After transient transfection with the NICD1 vector for 6 h, Hep3B and SMMC7721 cells were incubated in the presence or absence of TQ (60 μM) for 48 h. The cells were then subjected to *in vitro* MTT assays and WB analysis. Results showed that forced overexpression of NICD1 reversed the inhibitory effects of TQ on cell growth. MTT assay results in control, TQ, pIRES-NICD1 and TQ+pIRES-NICD1 groups were 0.9 ± 0.1, 88.15 ± 9.48, 43 ± 3.81, and 57.5 ± 10.1 in Hep3B cells, respectively, while corresponding values were 0.8 ± 0.2, 61.79 ± 9.35, 29.7 ± 4.1, and 40.8 ± 4.75 in SMMC7721 cells, respectively (Fig. 5C). WB revealed that forced NICD1 overexpression caused attenuation of TQ's inhibitory effects on expression of Notch1, Jagged1, Hes1, cyclinD1, CDK2 and Bcl-2, as well as on TQ-induced upregulation of p21 and Bax (Fig. 5D). These results suggest that TQ inhibits tumor cell growth via cell cycle arrest and apoptosis, induction at least in part due to repression of Notch signaling.

### TQ inhibits the growth of hepatocellular carcinoma *in vivo*

**Figure 6:**
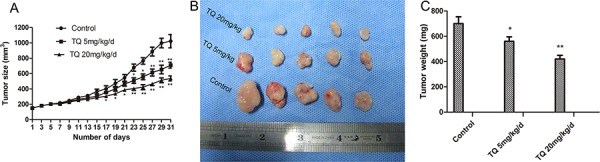
TQ inhibits tumor growth derived from Human Hep3B hepatocellular cells in nude mice Tumor-bearing mice were divided into three groups when volume of xenografts reached ~150 mm3. They were given s.c. injection of solvent, 5 mg/kg and 20 mg/kg TQ daily, respectively. **A.** Tumor volume and size was measured every two days as described in materials and methods; points, mean tumor size (*n* = 5); bars, SE. *p < 0.05 and **p < 0.01 compared with the control group. **B.** Macroscopic appearance of xenografts after TQ treatment for 31 days. **C.** Tumor weight of tumors after resection.

**Figure 7:**
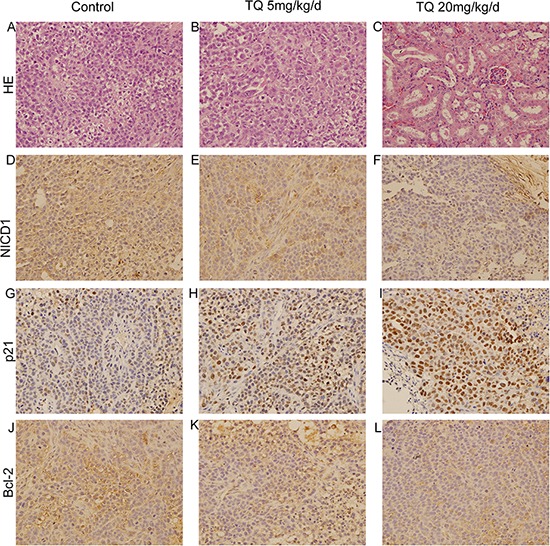
The anti-cancer effects of TQ on HCC *in vivo* H&E staining in control group **A.** and 5 mg/kg TQ treated group **B.** Influence of TQ on kidney epithelial cells in 20 mg/kg TQ treated group **C.** by H&E method. Immunohistochemical detection of NICD1, p21 and Bcl-2 at x200 magnification in the control, 5 mg/kg and 20 mg/kg TQ treated groups were demonstrated in **D, E** and **F, G, H** and **I, J, K** and **L**, respectively.

In order to verify our *in vitro* observation and to evaluate the anti-tumor efficacy of TQ *in vivo*, we analyzed its influence on the growth of liver tumor xenografts in athymic nude mice. TQ (5 mg/kg/d and 20 mg/kg/d) was administered subcutaneously (s.c.) beginning when Hep3B tumors reached 150 mm^3^. As shown in Fig. 6A, TQ treatment caused a statistically significant tumor volume decrease *vs*. the control group (*p* < 0.01) at day 31: the average tumor volumes in TQ-treated (5mg/kg/d or 20 mg/kg/d) *vs*. solvent-treated control mice were 710 ± 37.6 or 537 ± 32.5 cm^3^ *vs*. 1022.3 ± 387.1 cm^3^, respectively (*p* < 0.05). There was no statistical difference in average body weights (22.6 ± 2.9 g in the 5mg/kg/d group and 21.1 ± 3.2 g in the 20mg/kg/d group *vs*. 23.5 ± 4.1 g in controls), indicating that TQ exerted no obvious deleterious side effects in tumor-bearing mice. Fig. 6B-6C shows that tumor sizes and weights in TQ-treated mice were significantly smaller than in solvent-treated mice. In addition, H&E staining of the control and 5 mg/kg/d TQ-treated groups confirmed that tumor xenografts had been successfully established in both groups (Fig. 7A-7B). H&E staining of kidney epithelial cells in the 20 mg/kg/d TQ-treated group revealed normal histology (Fig. 7C), further establishing that TQ exerted minimal toxicity. We next investigated whether TQ-inhibited tumor growth in mice was associated with inactivation of the Notch pathway and expression of p21 and Bcl-2, as in cultured cells. Immunohistochemical staining of tumor tissue revealed that TQ treatment at the doses of 5mg/kg and 20mg/kg increased the expression of p21 protein, while this treatment inhibited the expression of NICD1 and Bcl-2 *vs*. controls (Fig. 7D-7L). Overall, these observations demonstrate that TQ inhibits the growth of HCC cells both *in vitro* and *in vivo*, associated with reduced activity of the Notch signaling pathway and induction of cell cycle arrest and apoptosis.

## DISCUSSION

Herbs contain potent bioactive natural substances, which are commonly used to treat a variety of diseases including rheumatological disorders [26] and breast cancer [27–28]. TQ extracted from Nigella sativa has been used by multiple human societies for centuries, and this substance has shown anti-tumor activity against lymphoblastic leukemia, breast cancer, prostate, and pancreatic cancer cells [10–13]. However, its effect and underlying molecular mechanisms in HCC have not been previously clarified. Our studies reveal, to our knowledge for the first time, that TQ induces anti-proliferative and apoptotic effects in HCC *in vitro* and *in vivo*, at least in part by inhibiting the Notch pathway. Based on our findings that TQ inhibits growth of cancer cells without conspicuous toxicity, this natural substance represents a promising new agent for the treatment and/or prevention of liver cancer.

Regulation of cell proliferation and differentiation is closely associated with activation of cell cycle-dependent cyclins, cyclin-dependent kinases (CDKs), and CDK inhibitors. The D-type cyclins with their matched kinase partners result in G1 progression, while cyclin E interacts with CDK2 and plays a significant role in cell cycle progression past the G1 phase [29]. p21, a key CDK inhibitor, can inactivate the cyclin/CDK complex, causing efficient cell cycle arrest [30]. In this study, we found that TQ effectively blocked G1-phase HCC cells from entering S-phase. Furthermore, our data illustrated that TQ suppressed the expression of cyclinD1 and CDK2, while simultaneously boosting p21 levels, which correlated with G1-phase arrest and growth suppression. According to reports by Shin [31], TQ induces cell cycle arrest by increasing p21.

As is known, cancer development and progression are attributed to destruction of the balance between cell proliferation and apoptosis [32]. Apoptosis is mediated by several cascade proteins, especially members of the Bcl-2 family and Bax [33]. Bcl-2 is an anti-apoptotic protein, while Bax (Bcl-2 Antagonist X) is a pro-apoptotic protein with the ability to antagonize Bcl-2, thereby increasing the activation of caspases, altering mitochondrial permeability, and triggering mitochondrial pathway apoptosis [34]. In the present study, we observed that TQ treatment of HCC cells increased Bax expression and diminished Bcl-2 expression. These expression alterations of Bax and Bcl-2 highlight a high Bax/Bcl-2 ratio, which is a significant factor in determining susceptibility to apoptosis: high Bax/Bcl-2 ratios resulted in a higher proportion of apoptotic cells than in control groups. These results establish that the apoptosis-promoting effect of TQ on Hep3B and SMMC7721 cells was at least in part due to modulation of Bcl-2 and Bax.

The Notch pathway is genetically altered in many hematopoietic and solid tumors, resulting in activation or repression of this pathway in the context of other oncogenic signaling pathways [35]. Many studies [21–24, 36–39] have confirmed that Notch can function as either an oncogene or a tumor suppressor gene, manifesting oncogenic roles in chronic lymphocytic leukemia (CLL), lung adenocarcinoma, and colorectal cancer, but tumor-suppressive activity in acute myeloid leukemia (AML), lung squamous cell carcinoma, and cutaneous squamous cell carcinoma. However, whether activated Notch inhibits or promotes HCC cell growth is still controversial. Qi *et al.* found that activated NOTCH1 represses liver cancer cells growth by the induction of cell cycle arrest and apoptosis [40], suggesting that Notch functions as a tumor suppressor in the liver. In contrast, two research groups revealed that the activated intracellular domain of Notch1 [41] or Notch2 [42] induces liver cancer development. In accordance with the reported conclusion of Villanueva *et al*. [41], our result demonstrated that levels of Notch1 mRNA expression in HCC cell lines were higher than in normal liver cells. Previous reports [43–44] showed that Notch2 or Notch3 are involved in gastric carcinogenesis or lung cancer progression. We found no statistically significant differences in Notch2 or Notch3 expression between HCC cells and normal liver cells (Fig. S1, S2); therefore, we did not choose Notch2 or Notch3 for further studies. To further investigate the possible molecular mechanism of TQ in the inhibition of HCC growth, we measured protein levels of Notch1, NICD1, Jagged1 and Hes1 in HCC cells. Our results indicated that expression levels of Notch1, NICD1, Jagged1 and Hes1 were markedly lower in TQ-treated cells than in control cells.

Furthermore, we observed that forced overexpression of NICD1 reversed the inhibitory effects of TQ on HCC cell proliferation. Our results also confirmed that TQ inhibited protein expression of Notch1 (receptor), Jagged1 (ligand), NICD1 (nuclear transcriptional factor), and Hes1 (downstream Notch pathway target gene) after transfection of a NICD-expressing vector, in agreement with our observed effects of TQ on HCC cells before transfection, suggesting that TQ attenuates Notch pathway activity. TQ also suppressed cyclinD1, CDK2 and Bcl-2, while blocking upregulation of p21 and Bax in Hep3B and SMMC7721 cells after NICD transfection. These results suggest that TQ inhibits the expression of cyclinD1, CDK2 and Bcl-2 while accelerating the expression of p21 and Bax, at least in part via repression of Notch signaling. A number of studies have shown that activated Notch can modulate expression of p21 and Bcl-2 [45–46], suggesting that p21 and Bcl-2 are downstream of TQ- induced Notch inhibition, and suggesting that TQ may restore normal proliferation and apoptosis by downregulating the Notch pathway.

γ-secretase is a proteolytic enzyme complex that contains transmembrane domains, comprised of a catalytic subunit (Presenilin-1 (PS-1) or PS-2) and accessory subunits (Aph1, Pen-2 and nicastrin) [17]. Inhibition of γ-secretase (GSI) blocks NICD, which travels into the nucleus, where it activates the expression of downstream target genes [18–19]. We determined the percentage of apoptotic cells after treatment with TQ 40 μM, GSI (1 μM), or TQ+GSI for 48 h. Apoptotic cells numbered (75.13 ± 7.1)% after 40 μM TQ treatment in Hep3B cells, (4.32 ± 1.27) in control group, (51 ± 4.6)% in 1 μM GSI-treated Hep3B cells, and (99 ± 0.5)% in the TQ+GSI treatment group (Fig. S3). We could not perform further research with GSI because we had already reached nearly 100% apoptosis in the combined treatment group.

Finally, our *in vivo* studies suggested that TQ inhibits tumor formation in xenografted mice by suppressing Notch-induced cell cycle arrest and apoptosis.

Several studies have illustrated the role of TQ in cancer metastasis. Khan *et al*. reported that TQ inhibits metastasis of MDA-MB-435 breast cancer cells by decreasing epithelial-to-mesenchymal transition (EMT) [47]. Siveen *et al*. found that TQ enhances chemosensitization and anticancer activity of Bortezomib in human multiple myeloma cells and mouse xenografts via inhibition of the NF-κB pathway [48]. It has reported that TQ represses tumor growth by modulating p38, STAT-3, PPAR-γ and other signaling pathways. [11, 49–52]. Hence, these events may also be responsible for TQ-induced inhibitory effects on the growth of HCC xenografts mice in our present study. It would be interesting to evaluate whether TQ inhibits these other pathways in future studies.

In summary, our study clarifies the inhibitory effect of TQ on growth of HCC cells *in vitro* and *in vivo*. Moreover, cell cycle arrest and apoptosis induced by TQ may be attributed, at least in part, to repression of the Notch signaling pathway. We have presented evidence showing that cell cycle arrest and apoptosis associated with Notch pathway inhibition are mechanisms by which TQ can inhibit HCC growth. These observations suggest that TQ should be considered as a promising agent for the prevention or treatment of HCC.

## MATERIALS AND METHODS

### Reagents

Thymoquinone was purchased from Sigma-Aldrich Co. (St. Louis, MO, USA). 3-(4,5-Dimethylthiazol-2-yl)-2,5-diphenyltetrazolium bromide (MTT) and Dimethyl sulfoxide (DMSO) were provided by Sigma Chemicals Co. (St. Louis, MO, USA). Primary antibodies against Notch1, NCD1, Jagged1, Hes1, Bcl-2, and Bax were ordeded from Abcam (Cambridge, MA, USA). Anti-p21, anti-cyclinD1, anti-CDK2 and anti-β-actin were obtained from Cell Signaling Technology (Danvers, MA, USA). Fetal bovine serum (FBS) was purchased from Gibco-BRL (Rockville, MD, USA). Dulbecco's Modified Eagle's Medium (DMEM) was ordered from Life Technologies (Carlsbad, CA, USA).

### Cell culture

The human HCC cell lines (Hep3B, SMMC7721, HepG2, Bel7402, MHCC97-L, MHCC97-H and HHCC) and normal liver cell line (L-02) were ordered from the Shanghai Institutes of Biological Sciences. Cells were grown in DMEM supplemented with 10% FBS, 100 U/mL penicillin and 2.5 mmol/L glutamine. All cells were cultured in a humidified incubator with 5% CO2 at 37°C.

### Cell viability assay

We used MTT assay to detect live cells as per manufacturer's instructions. In Brief, Hep3B, SMMC7721, HepG2, Bel7402, MHCC97-L, MHCC97-H and HHCC cells were seeded in 96-well plates at a density of 6000 per well, incubated in in appropriate medium for 24 to 48 h, then treated with ascending concentrations of TQ (20–80 μmol/L) for different time periods (24, 48 and 72 h). Cells were subsequently cultured d in medium with 0.5 mg/ml MTT for another 4 h. Following removal of the supernatant and adding 150 μl DMSO, the optical density(OD) at 490nm was determined by ELISA reader (Bio-Rad, Hercules, CA, USA). The inhibition rate (IR) of cell growth was calculated by formula: mean value of (1-treated group/control group) × 100%.

### Quantitative real-time RT-PCR

Total RNA was isolated from the cultured cells with the RNeasy Mini kit (Invitrogen, CA, USA) according to the manufacturer's instructions. cDNA was synthesized using SuperScript III First-Strand Synthesis Systemkit (Invitrogen). Quantitative real-time PCR (qRT-PCR) was carried out (Bio-Rad Laboratories, CA, USA) using SYBR *Premix Ex Taq* II (Tli RNase H Plus) (TaKaRa, Japan). The specific primers were as follows:Notch1, forward 5′-CCGCAGTTGTGCTCCTGAA-3′and reverse 5′-ACCTTGGCGGTCTCGTAGCT-3′; β-actin, forward 5′-CTCTTCCAGCCTTCCTTCCT-3′ and reverse 5′-AGCACTGTGTTGGCGTACAG-3′. The housekeeping gene β-actin was used as an internal control. The analyses of Notch1 data were according to the 2^−ΔΔCt^ mVethod.

### Expression plasmid construction and cell transfection

The full coding region that encodes human NICD1 (GenBank accession No. NM_017617.3) was amplified by PCR using primers (forward 5′-CTCGAGAATATGGTGCTGCTGTCCCGCAAG-3′ and reverse 5′-GGATCCGCACACAGACGCCCGAA GG-3′) from cDNA of U251 glioma cells [53]. The product was cloned into the pGEM-T Easy Vector (Promega, WI, USA) and conducted sequence analysis. The correct NICD1 cDNA subsequently was cloned into XhoI and BamHI sites of the bicistronic expression vector pIRES2-EGFP (enhanced green fluorescent protein) (Clontech Inc, Palo Alto, CA), allowing for translation of both target genes and the EGFP, and generated pIRES2-NICD1-EGFP. For transient transfection experiments, the cells were cultured 24 h before transfection in a 6-cm dish at a density of 2 × 10^5^ cells per dish. Transfecttion of the cells was performed using Lipofectamine 2000 (Invitrogen, CA, USA) with pIRES-NICD1-EGFP vector (pIRES-NICD1) and pIRES-EGFP empty vector (pIRES-EGFP) (as a negative control) according to the manufacturer's protocol.

### Cell cycle distribution analysis

Cells were incubated in 6-well plates at a density of 1.6 × 10^6^ cells per well, and dealt with various concentrations of TQ for 48 h when grown to 60% confluence. The cells from each well were collected and fixed with 70% ethanol overnight, following suspended by 0.5 ml PI (propidium iodide, Sigma-Aldrich) staining solution (50 μg/ml PI, 0.2% TritonX-100 in PBS and 100 μg/ml RNase) for half an hour at room temperature in dark place. Cell cycle distribution was determined by a FACS Calibur flow cytometer and the CellQuest software (Becton-Dickinson). All of the cell cycle experiments were conducted in triplicate.

### Assessment of cell apoptosis

Quantitative analysis of apoptosis was valuated using FITC Annexin V Apoptosis Detection Kit (Joincare Biosciences, Zhuhai, China) according to the manufacturer's directions. Tumor cells were cultured at a density of 1.6 × 10^6^ cells per well into 6-well plates, then were treated with indicated concentrations of TQ (0, 20, 40, 60 and 80 μM) and incubated for 48 h. Cells were resuspended by 500 μl 1× Annexin-binding buffer and stained with 5 ul AnnexinV-FITC, followed addition of 10 μl PI for 15 min. The percentage of apoptotic cells was analyzed by FACS Calibur flow cytometer.

### Western blot analysis

Western blot analysis was performed as previously described [54]. Protein extraction was prepared from TQ-treated and control group cells cultured in 6-cm dishes. The cells were harvested and lysed in lysis buffer. The protein (40–60 μg) was separated by 10% SDS-PAGE and transferred onto polyvinylidene difluoride (PVDF) membranes (Millipore, MA, USA) after its quantification. The membranes were blocked with 5% non-fat milk in tris-buffered saline with Tween (TBST) at 37°C for 2 h, subsequently were incubated with the primary antibodies overnight at 4°C, Horseradish peroxidase-linked anti-rabbit IgG (1:3,000) was used as the secondary antibody, then incubated at room temperature for 4 h. The antigen-antibody complexes were detected by enhanced chemiluminescence kit (Millipore, MA, USA). The band density was measured using Image Lab 4.0 (Bio-Rad) imaging software. Expression levels of the protein are assessed by a densitometric ratio of the targeted protein to the β-actin housekeeping protein.

### Human liver tumor xenograft mouse model

Four-week-old male athymic nude mice (BALB/c), weighing 18–22 g, were purchased from Shanghai Laboratory Animal Center (Shanghai, China) and maintained under specific pathogen-free conditions in accordance with Laboratory Animal Centre of Xi'an Jiaotong University animal care guidelines. Use and treatment of the animal were approved by the Institutional Animal Care and Use Committee of Xi'an Jiaotong University. Hep3B cells (2 × 10^6^) were injected s.c. into one flank. Tumor size and body weight was measured every other day. Tumor volume (V) was calculated with the formula V = (L × W^2^) × 0.5 where L is the length of the tumor and the W is the width. When a volume of xenografts reached ~150 mm^3^, 15 tumor-bearing mice were randomly divided into the following 3 groups(*n* = 5 in each group): control group given solvent (mixture of PBS/cremophor/ethanol, 5:3:2), TQ 5 mg/kg/d group, TQ 20 mg/kg/d group [12, 55]. When the tumor volumes of the control group reached ~1,100 mm^3^ (on day 31), the experiment was ended and the mice were sacrificed. Tumor tissue was immediately removed and fixed in 4% paraformaldehyde.

### Immunohistochemistry

The study was approved by the Research Ethics Committee of Xi'an Jiaotong University. HCC samples from mice xenograft were examined immunohistochemically by treptavidin-peroxidase (SP) method, as described previously [56]. Briefly, paraformaldehyde-fixed paraffin embedded tissues were cut into 4-μm sections. The sections were dewaxed, dehydrated, following antigen retrieval in a pressure cooking for 5 min then incubated with 0.3% (v/v) hydrogen peroxide for 10 min to block non-specific antigens and any endogenous enzyme. The sections were incubated with normal goat serum for 10 min then with the primary antibody against NICD1, P21 or Bcl-2 at 4°C overnight. The slides were washed with PBS and incubated with biotinylated goat anti-rabbit IgG (1:100 dilution; Zhongshan Golden Bridge Biotechnology, Beijing, China) for 20 min as the secondary antibodies. Each slide was colored with DAB (Sigma, St. Louis, MO, USA) in a dark room before incubated with peroxidase-conjugated streptavidin (Zhongshan Golden Bridge Biotechnology) for 10 min. At last, all the sections were rinsed with running water, counterstained by hematoxylin and dehydrated in graded ethanol. The slides were read and marked by two independent pathology experts under a microscope (Olympus Optical Co, Tokyo, Japan). Haematoxylin and eosin (H&E) staining was performed according to regular procedures.

### Statistical analysis

All experiments were repeated independently at least 3 times. Data are presented as the means ± SEM. Statistical comparisons were analyzed using one-way ANOVA or the Student's *t*-test. Differences were considered to indicate statistically significant with *P* < 0.05. All tests and *p*-values were two-sided. Statistical analysis was carried out using SPSS20.0 software (SPSS Inc. Chicago, USA).

## SUPPLEMENTARY FIGURES


