# Supplementary material for: TQ inhibits hepatocellular carcinoma growth in vitro and in vivo via repression of Notch signaling
Source: Oncotarget. 2015 Sep 21;6(32):32610–21. doi: 10.18632/oncotarget.5362 (PMC4741716; doi:10.18632/oncotarget.5362)
Supplement: Supplementary file 1 [file oncotarget-06-32610-s001.pdf]

SUPPLEMENTARY FIGURES

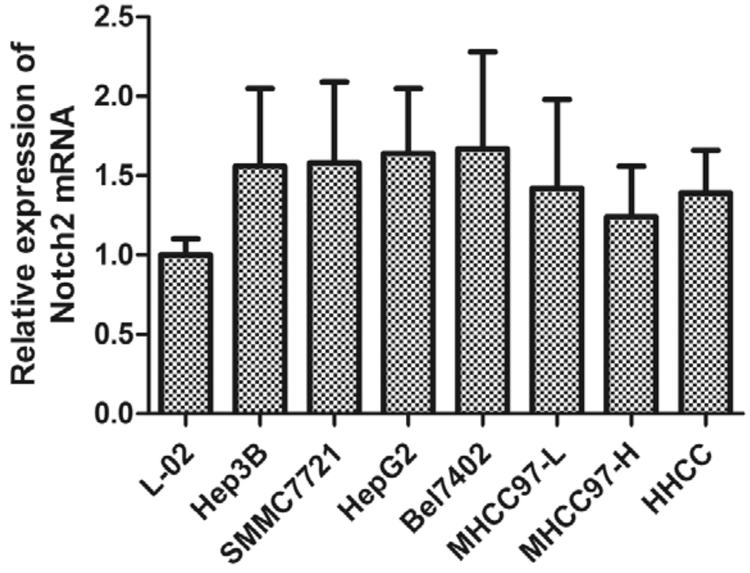

Supplementary Figure S1: Notch2 mRNA expression levels in seven different HCC cell lines and a normal liver cell. Determined by qRT-PCR analysis (mean ± SD, *n* = 3)

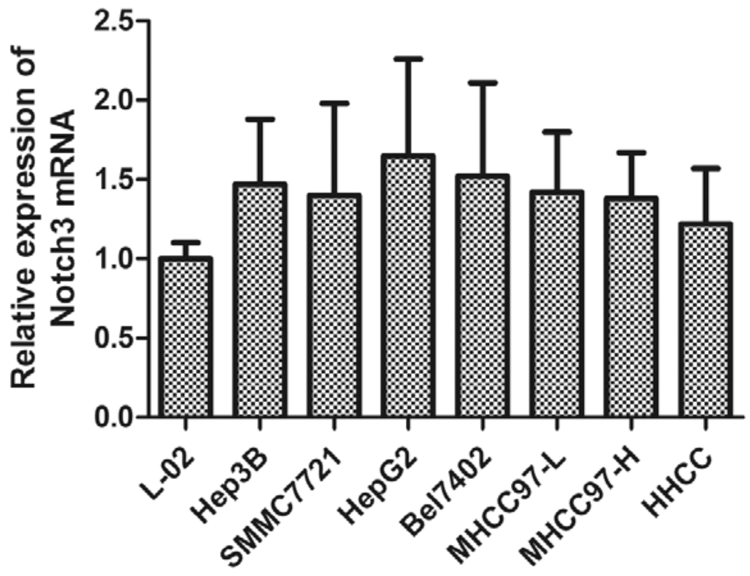

Supplementary Figure S2: Notch3 mRNA expression levels in seven different HCC cell lines and a normal liver cell. Determined by qRT-PCR analysis (mean ± SD, *n* = 3)

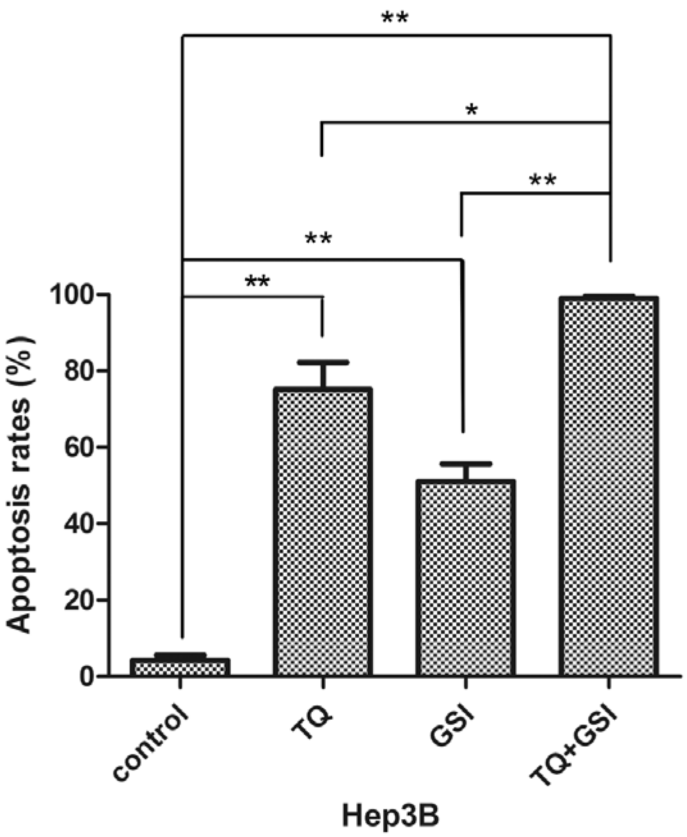

Supplementary Figure S3: Effects of 40 µM TQ and 1 µM GSI treatment on apoptosis in Hep3B cells. Percentage of apoptosis in each group were assessed by flow cytometry. All experiments were performed in triplicate and data are expressed as means ± SD (n = 3). Error bars represent SD of replicate data points. *$p < 0.05$, **$P < 0.01$.
